# Supplementary material for: Application of Hierarchical Dissociated Neural Network in Closed-Loop Hybrid System Integrating Biological and Mechanical Intelligence
Source: PLoS One. 2015 May 19;10(5):e0127452. doi: 10.1371/journal.pone.0127452 (PMC4437899; doi:10.1371/journal.pone.0127452)
Supplement: S1 Text — (DOC) [file pone.0127452.s001.doc]

Application of dissociated hierarchical neural network in closed-loop hybrid system integrating biological and mechanical intelligence

1. Yongcheng Li^1¶^, Rong Sun^2¶^, Bin Zhang^2&^, Yuechao Wang^1&^ and Hongyi Li^1&*^
2. ^1^ State Key Laboratory of Robotics, Shenyang Institute of Automation, University of Chinese Academy of Sciences, Shenyang, Liaoning, P. R. China
3. ^2^ Hefei National Laboratory for Physical Sciences at the Microscale, Hefei, Anhui, P. R. China
4. ^*^ Corresponding author
5. E-mail: [hli@sia.cn](mailto:hli@sia.cn) (HY)
6. ^¶^These authors contributed equally to this work.
7. ^&^These authors contributed equally to this work.

## Abstract Neural networks are considered the origin of intelligence in organisms. In this paper, a new design of an intelligent system merging biological intelligence with artificial intelligence was created. It was based on a neural controller bidirectionally connected to an actual mobile robot to implement a novel vehicle. Two types of experimental preparations were utilized as the neural controller including ‘random’ and ‘4Q’ cultured neurons artificially divided into four interconnected parts. . Compared to the random culture, the ‘4Q’ cultures presented absolutely different activities, and the robot controlled by the ‘4Q’ network presented better capabilities in search tasks. Our results showed that neural cultures could be successfully employed to control an artificial agent; the robot performed better and better with the stimulus because of the short-term plasticity. A new framework is provided to investigate the bidirectional biological-artificial interface and develop new strategies for a future intelligent system using these simplified model systems.

## Intro****du****ction

Although artificial intelligence has been investigated since 1960s, many problems associated with recognition, cognition, evolution, learning and memory still remain unsolved. Lack of plastic developmental phase was one of the reasons cited. However, this process has been discovered in the biological nervous system , with indications of positive performance . . The plastic developmental phase can be observed within a wide range of different temporal and spatial scales in biological systems. Therefore, differential plasticity has resulted in the development of different biological-mechanical hybrid systems in order to create more powerful and effective intelligence systems addressing this issue.

In the recent past, several different hybrid systems have been developed, consisting of human brains , living animal nervous systems , and dissociated neural cultures coupled to different robot systems. We focused our attention on robot systems coupled to the dissociated neural cultures because not only can robots be perfectly controlled to execute the task, but also the principles of information transfer within the biological neural network can be easily observed in these simple preparations.

Previous neuro-robot studies have shown that dissociated neurons were generally cultured by a polydimethylsiloxane (PDMS) ring to grow them into an isolated random network, which has always limited the abilities to process the complex information input. Several types of dissociated neural networks, which were cultured with a unique PDMS shape in order to develop a multilevel structure to address this limitation , were discussed in previous works. These neural cultures were employed to study persistent activities of the neural network , connectivity among neurons and construct a new ‘logic’ device . These primeval investigations suggested a potential for hierarchical structure of the neural cultures to make decisions under complex conditions using the neuro-robot system.

The use of neuro-robot hybrid systems is limited. Although, the modular neural network with two parts interconnected has been employed to control a virtual robot in previous works , performance of the idealized robot persisted at low levels (many hits). The differences embodied in the robot’s performance (number of hits) between random and modular cultures remained insignificant. In Warwick’s work , the neural networks were connected to the actual robot to execute an ‘avoiding wall’ task, which was accomplished at a low performance level (one information input, 67% turning accuracy, and chance level 50%). Other works utilizing the actual robot did not allow the robot execute any task, and resulted in 42.86% correct turning in both directions (chance level 25%). Earlier works, including the Porter group , used a hybrid neural-algorithmic controller rather than a neural-centered type.

In order to develop a more effective neural controller that merged the biological intelligence with the mechanical intelligence, first we built a closed-loop hybrid robot system by interfacing a differential mobile robot with a population of dissociated neurons that was cultured on Micro Electrode Arrays (MEA). Subsequently, a PDMS template was developed to culture the ‘4Q’ hierarchical neural network, which contained a more complex hierarchical construction than previous networks. It resulted in a significant improvement in robot control compared with a random neural network. The differences embodied in the neural networks’ functions and robot’s performance between random and ‘4Q’ cultures were significant and will be detailed in this paper, respectively. The robot system was able to complete the searching object’s task via the control of the dissociated neural network, and it eventually achieved percentages of correct turning at over 80% in both directions (chance level 25%) within the ‘4Q’ cultures. Surprisingly, without tetanus training, the performance of the robot system improved with the stimulus period in the experiments, providing possible evidence of plasticity change in the neural assemblies following feedback from an external environment.

## Materials and methods

### *Ethics Statement*

This study was carried out in strict accordance with the recommendations in the Guide for the Care and Use of Laboratory Animals. The animal experimental facilities were approved by the Anhui Provincial Department of Science and Technology [Approval ID: SYXK (Anhui) 2005-004]. All protocols involving animal work were approved by the Ethics Committee of Animal Experiments of the University of Science and Technology of China (Permit Number: USTCACUC1201051).

All surgery was performed under anesthesia. The adult pregnant rats were anesthetized with 99% carbon dioxide. After death by cervical dislocation, we removed the embryo from the uterus of the pregnant rat. The neurons were directly extracted from the hippocampus of embryo. All efforts were made to minimize animal suffering.

### *Culture preparation*

Low dense cultures of embryonic rat hippocampus were prepared as previously described with minor modifications. Briefly, hippocampi were removed from embryonic day 17–18 (E17–18) rats and were treated with trypsin for 15 min at 37°C, followed by washing and gentle trituration. Dissociated cells were plated on MEA chips at a density of 50-80 cells per mm^2^. MEA chips were coated with poly-L-lysine to facilitate cell adherence. Neurons growing near the electrodes formed a relatively isolated island using PDMS rings. The culture medium comprised DMEM (BioWhittaker) supplemented with 10% heat-inactivated bovine calf serum (HyClone), 10% Ham’s F-12 with glutamine (Bio-Whittaker), 50 units ml penicillin streptomycin (Sigma), and 1×B-27 supplement (Invitrogen Gibco). Twenty-four hours after plating, one-third of the culture medium was replaced with a fresh medium supplemented with 20 mM KCl. Cytosine arabinoside (Sigma) was added to the culture dish (final concentration, 5 µM) around 7–10 days *in vitro* (div) to prevent overgrowth of glial cells. Cultures of 14 to 20 days were used in the experiments.

### *Neuro-robot architecture*

The overall closed-loop system consisted of two parts including the main PC and slave PC components (Fig. 1).

The hardware of main PC included the MEA device, where electrical signal was recorded by a MEA1060-Inv-BC amplification system (Multichannel Systems, MCS, Reutlingen, Germany). The home-made stimulating hardware (illustrated in Fig. 2A) provided electrical stimulation using the arbitrary waveform to 60 electrodes on the MEA in real time. It was a workstation directly linked to a DAQ data acquisition card for conducting computationally expensive neural data analysis. A pair-pulse electrical stimulation with an interval time of 50 milliseconds and an amplitude ±230 mv (negative first, duration was 1.5 milliseconds) was provided to neural network to serve as coding information input.

The software of the main PC component was modified from the toolbox written by Daniel Wagenaar (http://www.danielwagenaar.net/res/software/meabench/) and it ran on the directly linked workstation in a Linux operating system. This modified software, which retained all the functions of the toolbox, also added new functions to control all the parameters of the stimulation and performed the required data processing, such as communication with the robot control system, the implementation of the coding/decoding, spike detection, artifact filter, and short- term plasticity schemes.

The hardware of the slave PC consisted of a separate workstation running the robot control interface along with a robot system that included a two-wheel differential robot and a camera. Software installed in the separate workstation was programmed into a GUI interface to control the robot. As illustrated in Fig. 2B, this GUI monitored the position of the robot and objects in real time, controlled the motion of the robot manually, and displayed the data exchanged between the robot and the cultured neurons. Various components of architecture were communicated via TCP/IP sockets. This design allowed distributed processing of the computational loads via multiple machines throughout the internal network.

### *‘4Q’ culture*

An SU-8 mold was utilized to create a mask in order to culture the ‘4Q’ neural network hierarchy. The SU-8 mold was fabricated by UV lithography (Fig. 3A). It consisted of a two-layered SU-8 photoresist (MicroChem). The first layer SU-8 3005 photoresist was spin-coated on the cleaned Si wafer to a thickness of 10µm. The coated wafer was baked on the hot plate at a temperature of 95°C for 2min (soft bake). The SU-8 3005 photoresist was exposed by UV light under the photo mask containing a microchannel. After UV exposure, the coated wafer was baked on the hot plate at a temperature of 95°C for 2min (after exposure bake). Next the exposed SU-8 photoresist was developed by a SU-8 developer. After finishing the first layer, SU-8 2150 was spin- coated as a 150-µm-thick second layer and soft baked at 95°C for 30min. The chamber photo mask was aligned to the microchannel patterned on the wafer followed by UV exposure, and baked again at 95°C for 15min. Finally, the SU-8 2150 wafer was created by the SU-8 developer.

The mixture of PDMS pre-polymer and curing agent (10 : 1 (w/w), Dow Corning Corp) was poured on the SU-8 mold. PDMS was cast on the SU-8 mold and cured on the hot plate at 80°C for 40min. After curing, the PDMS device was removed from the SU-8 mold and attached to the MEA platform (Fig. 3B and 3C). Subsequently, low density cultures of embryonic rat hippocampus were prepared to produce the dissociated ‘4Q’ neural network (Fig. 4A and 4B).

### *Experiment protocol*

The typical experimental protocol included the following steps:

1. The dissociated neural cultures were transferred from the culture medium to the extracellular solution (ESC).
2. The I/O of areas was selected by supplying a stimulus to a set of electrodes
3. The robot was run for 15 minutes.
4. After resting for 2 to 4min, some of the robot parameters were optimized.
5. The step 3 and step 4 were repeated at least twice

During the first step, the spontaneous activities of the neural network were reduced by changing the culture medium to the ECS. During the second step of the experiment, the stimulus described above was delivered to each of the selected candidate electrodes every 30 seconds until at least two electrodes were identified to evoke the network firing. Empirically, two or more electrodes would be selected as the ‘input areas’ based on the difference in activities evoked by them, respectively. Areas that responded the most to stimulation from the corresponding input area were selected as the ‘output areas’, implying that one electrode/input area corresponded to one ‘output areas’. Every time one ‘output areas’ was confirmed, the remaining areas of neural network were defined as the corresponding ‘other areas’.

During step 3 and 4, the robot commanded by neurons moved freely from any corner of the arena to the objects in the middle of the arena. At least a 20-second interval was ensured between two adjacent stimulations due to the existence of the refractory period of the neurons. The trial was not stopped if the robot reached the objects, however, the robot would be resettled to the start position and the trial continued. Each trial ended after a 15-minute operation. This step was repeated at least twice in view of the hypothesis that the neural network dynamically modulated its activities to respond stably to external input along with the data constantly delivered to the neural network . We hoped the hypothesis would improve the robot’s performance. Finally, all the trajectories of the robot’s movements and activities of the dissociated cultures were recorded in the hard disk.

### *Statistical analysis*

We used a total of 14 different cultures, ranging from 12 to 20 DIV (days *in vitro*), including 6 random hippocampal cultures. The other 8 experiments used the hippocampal cultures, divided into four interconnected quadrants by a confinement device, as described above.

The Kolmogorov–Smirnov test (K–S test or KS test) was employed to assess the significant differences among diverse experimental conditions. The K-S test was used to evaluate the differences between two groups of independent samples. This non-parametric test was performed with a MATLAB program and P values of < 0.05 were considered significant.

## Results

### *Dissociated neural network with a hierarchical structure*

As illustrated in Fig. 4A and 4B, the neurons were cultured on the preparative MEA (Fig. 3C) to develop neural networks named ‘4Q’ cultures, with a hierarchical structure. The functional activities could be obtained by the MEA system from 14 DIV. Spontaneous activities were always observed synchronously throughout the network since the 4 quadrants were interconnected with the microchannels on the mask (Fig. 4C). However, the refined hierarchy of these cultures was activated under stimulation . This discovery will be further described in detailed in next section.

### *Network dynamics in the two neural networks*

An electric stimulation with an amplitude of ±230 millivolts was applied to the random and ‘4Q’cultures to evoke the activities of these neural networks. The neuron burst firing was also observed without any stimulation and lasted several seconds throughout the neural network. In both types of cultures, all the electrodes surrounding the growing neurons recorded their activities in the spontaneous condition of the neural network (top panels of Fig. 5A and 5B). During the spontaneous activity, differences in firing based on the hierarchical structure were easily observed. Most of the neurons in the neural network fired during every burst firing in random cultures, but often only part of the neurons in the neural network fired in ‘4Q’ cultures. Most of the electrodes recording the spontaneous activities showed a response to the stimulation in the random culture, but only some of these electrodes responded in the ‘4Q’ cultures because of the hierarchical structure of cultures (bottom panels of Fig. 5A and 5B). As illustrated in Fig. 6, this response specificity was increasingly apparent in the ‘4Q’ cultures than the random cultures. The network in the ‘4Q’culture employed various neural circuits to respond to different stimuli, but nearly the same circuit was used to respond to different stimuli in the random cultures.

To further assess the differences in response to the stimulation in these two kinds of culture, we calculated a ratio, by dividing the number of electrodes responding simultaneously to different stimuli with the number of total electrodes responding to the stimuli in one culture. A smaller ratio suggested a greater probability of different responses evoked by the neural network to different stimuli. We obtained the ratios from six random cultures and eight ‘4Q’ cultures (Fig. 5E), and a total of 10 ratios from the random cultures were compared to 10 ratios from the ‘4Q’cultures to evaluate their differences. As shown in Fig. 5E, ratios of the ‘4Q’ cultures were significantly smaller than those of the random cultures (P < 0.05, Kolmogorov- Smirnov Z test). It must be emphasized that the response specificity of the ‘4Q’ cultures was stronger than that of the random cultures.

The response specificity was presented more easily in the ‘4Q’ cultures than in the random culture. The evoked firing rates in the corresponding ‘output areas’ and ‘other areas’ were calculated, respectively, and were also compared with each other in both types of cultures. As illustrated in Fig. 5D, the firing rate in the ‘output areas’ was significantly larger than the one in the ‘other areas’ of the ‘4Q’ culture (P < 0.05, Kolmogorov- Smirnov Z test). However, this result was not observed in the random cultures (Fig. 5C). As previously mentioned, compared to previous random cultures, we demonstrated that the ‘4Q’cultures had completely different properties in signal propagation. This difference resulted in a significant improvement in the performance of the neuro-robot hybrid systems.

### *Comparison of the performance of robot systems with different neural controllers*

A closed-loop robot control was implemented in both types of cultures. All the parameters associated with robot movement and activities of the neural network have been recorded experimentally. Consistent with the results above (Fig. 5 and 6), the evoked activities showed a strong overlap regardless of the position of the stimulating electrodes in the random cultures. The percentages represented by the number of evoked electrodes divided by recording electrodes in the corresponding ‘output areas’ were 58%±22.3% for the random cultures and 62%±20.1% for the ‘4Q’ cultures. These percentages indicated that the movement of the robot was controlled under the correct evoked activities of the neural network. The results comparing the performance of the robot controlled by the random and ‘4Q’ cultures are illustrated in Fig. 7C and 7D. Time spent on finding the objects and correct turning percentage was employed to evaluate the performance of the robot systems. As shown in Fig. 7C and 7D, the time spent on finding the objects was less (Fig. 7D) and the number of correct turns of the robot controlled by the ‘4Q’ culture (Fig. 7C) was larger than in the random cultureFig.Fig.. These results further suggested that the hybrid system with the hierarchical cultures yielded a better performance. As illustrated in Fig. 7A and 7B, the hybrid system in the closed-loop robot controlled by the ‘4Q’culture Fig.could reach the objects within 400 seconds and performed 7 turns, which were all correctly executed by the robot. This result demonstrated that the performance of the neuro-robot system has been powerfully improved by the neural network constructed with a complex hierarchy.

### *Plasticity of the biological neural network in the neuro-robotic system*

Although the robot reach the objects under the control of the ‘4Q’ cultures during the first phase, errors still occurred and intermittently the robot took more time to reach the objects, especially in the random cultures. To address this issue, a strategy previously described in the experimental protocol was employed by providing the plasticity of the biological intelligence in the dissociated culture to the hybrid system. As illustrated in Fig. 8A, the performance of the robot controlled by the ‘4Q’ culture was improved when the experimental process was employed. In this example, time spent on finding the objects changed from 360 seconds, 316 seconds, to 279 seconds, and the correct turning ratios during this time were 67%, 75%, and 100%, respectively. The same performance of the robot was further assessed in Fig. 8B and 8C. The graphs in Fig. 8B showed the changes in time spent finding the objects in our hybrid system. However, the expected reduction due to the plasticity change was only observed in the ‘4Q’ cultures. A statistically significant difference was occurred between the first 15 minutes and the last 15 minutes (P < 0.05, Kolmogorov- Smirnov Z test). Another parameter evaluating the performance of the robot is illustrated in Fig. 8C. The obvious increase of the correct ratios bidirectionally along with the experimental process was observed in the ‘4Q’ cultures, but in only one of the random cultures. Interestingly, the correct ratios in two directions were counted, suggesting that the ‘4Q’ cultures simultaneously modulated themselves to adapt to two different information inputs (in fact, we obtained data, where the ‘4Q’ cultures only dynamically adapted to one of the two inputs like the random cultures). These preliminary analyses of changes in our hybrid system showed that the dissociated neural networks were able to dynamically ‘recognize’ the different stimulation inputs to strengthen the corresponding connections that were employed to transmit information by the ‘4Q’ culture. Statistically significant difference in the correct turning ratio was observed between the first 15 minutes and the last 15 minutes (P < 0.05, Kolmogorov- Smirnov Z test). The increase in correct turning ratio in both directions (along with reduced time) was present in some random cultures, but the fact that more cultures changed in one or none of the directions impacted the final statistical results. Therefore, despite the occurrence of plasticity in the random cultures it was not adequate to further improve the performance of the robotic system due to inherent limitation of the structure of the random cultures.

## Discussion

We have provided evidence that the employment of hierarchical cultures creates different neural circles and greatly reduces the appearance of network-wide synchronized bursts (Fig. 5 and 6), resulting in significant improvement in the robot’s performance (Fig. 7). Interestingly, the hierarchical network contributed to the plasticity of neural network even without tetanus training (Fig. 8). These results prompted us to increase the hierarchy of the network in an attempt to enable the dissociated cultures to process more complex behavioral information.

In our experiment, the robot’s movement was controlled by cultures via electrical stimulation that lasted about one hour. These short one-hour simulations were ideal for improving the robot’s performance by inducing plasticity change of the neural network. Based on the hierarchical culture of dissociated neurons, it is possible to stimulate initially during the beginning of neural network formation to functionally define the neurons, but not physically distinct sub-populations neurons. These stimulations will be used in future experiments within the same culture.

Though the plasticity developmental phase was eventually employed in our hybrid system, it took nearly one hour for a satisfying performance. In previous studies, a training strategy utilizing the tetanic and spatiotemporal stimulations was used and demonstrated effective improvements of the robot’s performance . Therefore, in order to effectively evoke the plasticity of the cultures, a more productive learning scheme needs to be designed. The spike-timing dependent plasticity (STDP) learning mechanism detailed in is a promising candidate. The application of this training phase will provide a useful method to investigate the mechanism of synaptic transmission and receptors involved in the process of adaptation and learning depending on specific stimulation protocols.

Our long-term research objective is to exploit neural plasticity in more complex hybrid systems to perform closed-loop experiments that measure the effective use of biological, computational and learning principles even with relatively simple neural preparations. The neuro-robot framework is also a valid tool for the study of mechanisms of neural coding, neural computation, self-organization and reinforced learning. This kind of architecture is a promising foundation that can be employed in the design of neural prosthetics, and can create fundamentally different types of artificial or hybrid intelligence.

## Conclusion

In this paper, issues related to bi-directional closed-loop interface involving cultures with more complex hierarchy than previously reported were introduced for the first time. Statistical comparisons obtained from the ‘4Q’ and random cultures were calculated to highlight the advantages of modular cultures and neural assemblies that are relevant to the performance of the actual artificial agent. The self-adaptive plasticity of the ‘biological brain’ in a robot system was observed and employed in our hybrid system. Finally, we successfully connected both types of dissociated neural networks originating in the hippocampus of embryonic rats to a mobile robot system bidirectionally. However, we found that the robot system controlled by the hierarchical neural network was more likely to adapt to the plasticity change evoked by stimulation, and was able to execute its task of finding the object at a higher performance level.

## Acknowledgment

The authors want to thank Prof. Guoqiang Bi and Prof. Pakming Lau at the University of Science and Technology of China. We appreciate their useful advice and support for the experimental setup.

## References

1. Wagenaar DA, Pine J, Potter SM (2006) An extremely rich repertoire of bursting patterns during the development of cortical cultures. BMC Neuroscience 7: 11.

2. Raichman N, Volman V, Ben-Jacob E (2006) Collective plasticity and individual stability in cultured neuronal networks. Neurocomputing 69: 1150-1154.

3. Izhikevich EM, Gally JA, Edelman GM (2004) Spike-timing dynamics of neuronal groups. Cerebral cortex 14: 933-944.

4. Xu S, Jiang W, Poo M-m, Dan Y (2012) Activity recall in a visual cortical ensemble. Nature Neuroscience 15: 449-455.

5. Patil PG, Turner DA (2008) The development of brain-machine interface neuroprosthetic devices. Neurotherapeutics 5: 137-146.

6. Di GJ, Gong W, Haburcakova C, Kögler V, Carpaneto J, Genovese V, et al. (2010) Development of a closed-loop neural prosthesis for vestibular disorders. Journal of Automatic Control 20: 27-32.

7. Rachmuth G, Shouval HZ, Bear MF, Poon C-S (2011) A biophysically-based neuromorphic model of spike rate-and timing-dependent plasticity. Proceedings of the National Academy of Sciences 108: E1266-E1274.

8. van Pelt J, Wolters PS, Corner MA, Rutten WL, Ramakers GJ (2004) Long-term characterization of firing dynamics of spontaneous bursts in cultured neural networks. Biomedical Engineering, IEEE Transactions on 51: 2051-2062.

9. Barkan U, Horn D (2006) Spatiotemporal clustering of synchronized bursting events in neuronal networks. Neurocomputing 69: 1108-1111.

10. Maeda E, Robinson H, Kawana A (1995) The mechanisms of generation and propagation of synchronized bursting in developing networks of cortical neurons. The Journal of Neuroscience 15: 6834-6845.

11. Shahaf G, Marom S (2001) Learning in networks of cortical neurons. The Journal of Neuroscience 21: 8782-8788.

12. Jarosiewicz B, Chase SM, Fraser GW, Velliste M, Kass RE, Schwartz AB (2008) Functional network reorganization during learning in a brain-computer interface paradigm. Proceedings of the National Academy of Sciences 105: 19486-19491.

13. Ikegaya Y, Aaron G, Cossart R, Aronov D, Lampl I, Ferster D, et al. (2004) Synfire chains and cortical songs: temporal modules of cortical activity. Science 304: 559-564.

14. Bi G-Q, Rubin J (2005) Timing in synaptic plasticity: from detection to integration. Trends in Neurosciences 28: 222-228.

15. Raichman N, Ben-Jacob E (2008) Identifying repeating motifs in the activation of synchronized bursts in cultured neuronal networks. Journal of Neuroscience Methods 170: 96-110.

16. Yanagisawa T, Hirata M, Saitoh Y, Kishima H, Matsushita K, Goto T, et al. (2012) Electrocorticographic control of a prosthetic arm in paralyzed patients. Annals of Neurology 71: 353-361.

17. Hochberg LR, Serruya MD, Friehs GM, Mukand JA, Saleh M, Caplan AH, et al. (2006) Neuronal ensemble control of prosthetic devices by a human with tetraplegia. Nature 442: 164-171.

18. Leuthardt EC, Schalk G, Wolpaw JR, Ojemann JG, Moran DW (2004) A brain–computer interface using electrocorticographic signals in humans. Journal of Neural Engineering 1: 63-71.

19. Santhanam G, Ryu SI, Byron MY, Afshar A, Shenoy KV (2006) A high-performance brain–computer interface. Nature 442: 195-198.

20. Schwartz A, Cui X, Weber D, Moran D (2006) Brain-Controlled Interfaces: Movement Restoration with Neural Prosthetics. Neuron 52: 205-220.

21. O’Doherty JE, Lebedev MA, Ifft PJ, Zhuang KZ, Shokur S, Bleuler H, et al. (2011) Active tactile exploration using a brain–machine–brain interface. Nature 479: 228-231.

22. Warwick K, Xydas D, Nasuto SJ, Becerra VM, Mark W. Hammond, Downes JH, et al. (2010) Controlling a Mobile Robot with a Biological Brain. Defence Science Journal 60: 5-14.

23. Novellino A, D'Angelo P, Cozzi L, Chiappalone M, Sanguineti V, Martinoia S (2007) Connecting Neurons to a Mobile Robot: An In Vitro Bidirectional Neural Interface. Computational Intelligence and Neuroscience 2007: 1-13.

24. Pizzi RMR, Rossetti D, Cino G, Marino D, A.L.Vescovi, Baer W (2009) A cultured human neural network operates a robotic actuator. Biosystems 95: 137-144.

25. Potter A, DeMarse TB, Bakkum DJ, Booth MC, Brumfield JR, Chao Z, et al. (2004) Hybrots: hybrids of living neurons and robots for studying neural computation. Proceedings of Brain Inspired Cognitive Systems: 1-5.

26. Bakkum DJ, Chao ZC, Potter SM (2008) Spatio-temporal electrical stimuli shape behavior of an embodied cortical network in a goal-directed learning task. Journal of Neural Engineering 5: 310-323.

27. Jun SB, Hynd MR, Dowell-Mesfin N, Smith KL, Turner JN, Shain W, et al. (2007) Low-density neuronal networks cultured using patterned poly-l-lysine on microelectrode arrays. Journal of Neuroscience Methods 160: 317-326.

28. Vishwanathan A, Bi G-Q, Zeringue HC (2011) Ring-shaped neuronal networks: a platform to study persistent activity. Lab on a Chip 11: 1081-1088.

29. Erickson J, Tooker A, Tai YC, Pine J (2008) Caged neuron MEA: A system for long-term investigation of cultured neural network connectivity. Journal of Neuroscience Methods 175: 1-16.

30. Feinerman O, Rotem A, Moses E (2008) Reliable neuronal logic devices from patterned hippocampal cultures. Nature Physics 4: 967-973.

31. Tessadori J, Bisio M, Martinoia S, Chiappalone M (2012) Modular neuronal assemblies embodied in a closed-loop environment: toward future integration of brains and machines. Frontiers in neural circuits 6: 1-16.

32. Lau P-M, Bi G-Q (2005) Synaptic mechanisms of persistent reverberatory activity in neuronal networks. Proceedings of the National Academy of Sciences of the United States of America 102: 10333-10338.

33. le Feber J, Stegenga J, Rutten WLC (2010) The Effect of Slow Electrical Stimuli to Achieve Learning in Cultured Networks of Rat Cortical Neurons. Plos One 5.

34. Bi G-q, Poo M-m (1998) Synaptic modifications in cultured hippocampal neurons: dependence on spike timing, synaptic strength, and postsynaptic cell type. The Journal of Neuroscience 18: 10464-10472.

## Figure legends

**Fig. 1. Framework of the neuro-robot system.** Two parts are included in this hybrid system:(i) Main PC components, employed to run the developed software tools, comprising the stimulator, the MEA coupled to live neurons, the amplification, and the data acquisition card; (ii) Slave PC, hosting the robot control software to command the robot system. The main PC communicates with the slave PC by a mean of network port running the TCP/IP protocol.

**Fig. 2. Home-made stimulator and the robot control software. A.** Implementation of the stimulator system including the pre-amplifier of the existing recording system. **B**. A graph user interface (GUI) employed to control the robot is written by MFC. The monitor in the left side of the GUI is able to display the position of the robot in real time; the buttons in the middle of the panel are employed to manually control the robot; and the right side of the panel can connect with the main PC and display the information from/to the main PC.

**Fig. 3. Manufacture of the ‘4Q’ cultures with the tailor-made PDMS. A.** Schematic of PDMS device fabrication. **B** Schematic of the PDMS device attached to 4Q MEA **C.** Physical map of the PDMS device attached to 4Q MEA.

**Fig. 4. Four-well neural network on the ‘4Q’ MEA. A.** Dissociated neurons coupled to MEA are cultured to ‘4Q’ shape with a PDMS. The white raised dots (marked by the arrow) are the cell bodies, and black dots are the electrodes. **B.** One of the four quadrants is zoomed in to illustrate more details of the culture. The light raised dots are the cell bodies. **C.** Raw electrode data of the ‘4Q’ culture are displayed. The 2 seconds of spontaneous activity in a total of 59 electrodes (electrode in row 5 column 1 is ground electrode) were acquired from a dissociated culture of 15 DIV. The augmented amplitudes recorded in these electrodes suggest occurrence of the firing spike. The dataset presented here is similar to Fig. 6C and 6D.

**Fig. 5. Evoked activity in random and ‘4Q’ hippocampal networks. A. Top.** Raster plot of the spontaneous activity exhibited by a random hippocampal culture (140 s of activity acquired from a dissociated culture of 14 DIV).**Bottom.** Raster plot of the evoked activity exhibited by the same random hippocampal culture (electrode 18 was stimulated. 2 seconds of activities evoked by the electrical stimulation are illustrated for each stimulation). **B. Top.** Raster plot of the spontaneous activity exhibited by a ‘4Q’ hippocampal culture (120 s of activity acquired from a dissociated culture of 15 DIV).**Bottom.** Raster plot of the evoked activity exhibited by the same ‘4Q’ hippocampal culture (electrode 16 was stimulated. 2 seconds of activities evoked by the electrical stimulation are illustrated for each stimulation). In A and B, the activity of 59 electrodes is displayed. Each small vertical bar represents a spike. Each line represents an electrode. **C.** Box-plot of the evoked spikes’ firing rate in the ‘output areas’ and the ‘other areas’ compartment with respect to stimulating electrodes in the random cultures. In the random network, No statistical differences were noted with 229 electrical stimulations in 6 cultures. **D.** Box-plot of the evoked spike firing rate in the ‘output areas’ and the ‘other areas’ compartment with respect to stimulating electrodes in the ‘4Q’ cultures. In the ‘4Q’ network, the evoked spike firing rate is statistically higher for the ‘output areas’ compartment; 257 electrical stimulations in 6 cultures. **E.** Box-plot of the percentage of redundant electrodes in the two stimulations (total of 20 datasets was calculated. Ten of those datasets were acquired from 6 random cultures, and others were from 8 ‘4Q’ cultures). Box range: percentile 25–75; box whiskers: percentile 5–95; line: median. Two sample Kolmogorov-Smirnov Z test for nonparametric test, significant differences are indicated by asterisks, and the significance level =*P < 0.05.

**Fig. 6. Activities evoked by a different stimulus in the random and ‘4Q’ cultures. A. B.** Raw electrode data of the random culture are displayed. The 2 seconds of activities evoked by the electrodes in the column 1 row 6 (A) and column 7 row 4 (B) in a total of 59 electrodes(electrode in row 5 column 1 is ground electrode) were acquired from a dissociated culture of 16 DIV. **C.D.** Raw electrode data of the ‘4Q’ culture are displayed. 2 seconds of activities evoked by the electrodes in the column 8 row 2 (C) and column 1 row 7 (D) in a total of 59 electrodes were acquired from a dissociated culture of 15 DIV. The augmented amplitudes recorded in these electrodes suggested occurrence of the firing spike.

**Fig. 7. Performance of the hybrid systems in the random and ‘4Q’ cultures. A.** Trajectory of the robot in a successful search task. The blue dot represents the location of the object, the red arrows represent the direction of the head of the robot, and the deep red dots represent the robot’s location, in which every time the neural network makes the decision. **B.** The detailed data analysis of the search task in A. from top to bottom, the first figure shows the distance between the object and the robot; the second figure shows the relative location between the object and the robot; the third figure shows the correct times of command executed by the robot and the X label represents the number of total commands from the beginning until now; the fourth figure shows the direction determined by the neural network. At the 0s time point, the robot has no action because the power is off. **C.** Box-plot of the correct turning percentage of the robot before reaching the object in both kinds of cultures, the percentage of the ‘4Q’ cultures is statistically higher. **D.** Box-plot of the time spent on reaching the object in both kinds of cultures, the time of spending in the ‘4Q’ cultures is statistically less, with 10 reaching in 3 random cultures and 12 reaching in 4 ‘4Q’ cultures. The datasets used in figure D are the same with C. Box range: percentile 25–75; box whiskers: percentile 5–95; line: median. Two-sample Kolmogorov-Smirnov Z test for nonparametric test, significant differences are indicated by asterisks, and the significance level =*P < 0.05.

**Fig. 8. Evolution of performance of the neuro-robot system. A.** Trajectories of the robot in three successful search tasks. The blue dot represents the location of the object, the yellow, red, and blue arrows represent the direction of the head of robot, and the yellow, light red , and deep red dots represent the robot’s location every time the neural network outputs the decision during three different time phases, respectively. **B.** Histogram of the time spent by the robot in reaching the object in both the cultures during the three time phases. Statistics of the time spent reaching the object during the three time phases are calculated (mean ± S.D). **C.** Histogram of the correct turning percentage of the robot in both types of culture. The statistics for the correct turning of the two directions during three time phases are calculated, respectively. Blue bar is left turning (mean ± S.D), and red bar is right turning (mean ± S.D).Two-sample Kolmogorov- Smirnov Z test, significant differences between two datasets are indicated by asterisks, and the significance level =*P < 0.05.

## Supporting Information Legends

**Text S1. A copy of this manuscript showing the changes.**
